# Plasma Metabolomics in Human Pulmonary Tuberculosis Disease: A Pilot Study

**DOI:** 10.1371/journal.pone.0108854

**Published:** 2014-10-15

**Authors:** Jennifer K. Frediani, Dean P. Jones, Nestan Tukvadze, Karan Uppal, Eka Sanikidze, Maia Kipiani, ViLinh T. Tran, Gautam Hebbar, Douglas I. Walker, Russell R. Kempker, Shaheen S. Kurani, Romain A. Colas, Jesmond Dalli, Vin Tangpricha, Charles N. Serhan, Henry M. Blumberg, Thomas R. Ziegler

**Affiliations:** 1 Nutrition and Health Sciences, Graduate Division of Biological and Biomedical Sciences, Laney Graduate School, Emory University, Atlanta, Georgia, United States of America; 2 Center for Clinical and Molecular Nutrition, Emory University School of Medicine, Atlanta, Georgia, United States of America; 3 Department of Medicine, Emory University School of Medicine, Atlanta, Georgia, United States of America; 4 National Center for Tuberculosis and Lung Disease, Tbilisi, Georgia; 5 Department of Civil and Environmental Engineering, Tufts University, Medford, Massachusetts, United States of America; 6 Center for Experimental Therapeutics and Reperfusion Injury, Harvard Medical School, Boston, Massachusetts, United States of America; 7 Atlanta Veterans Affairs Medical Center, Decatur, Georgia, United States of America; 8 Department of Epidemiology, Rollins School of Public Health, Emory University, Atlanta, Georgia, United States of America; University of Cape Town, South Africa

## Abstract

We aimed to characterize metabolites during tuberculosis (TB) disease and identify new pathophysiologic pathways involved in infection as well as biomarkers of TB onset, progression and resolution. Such data may inform development of new anti-tuberculosis drugs. Plasma samples from adults with newly diagnosed pulmonary TB disease and their matched, asymptomatic, sputum culture-negative household contacts were analyzed using liquid chromatography high-resolution mass spectrometry (LC-MS) to identify metabolites. Statistical and bioinformatics methods were used to select accurate mass/charge (*m/z*) ions that were significantly different between the two groups at a false discovery rate (FDR) of q<0.05. Two-way hierarchical cluster analysis (HCA) was used to identify clusters of ions contributing to separation of cases and controls, and metabolomics databases were used to match these ions to known metabolites. Identity of specific D-series resolvins, glutamate and *Mycobacterium tuberculosis (Mtb)*-derived trehalose-6-mycolate was confirmed using LC-MS/MS analysis. Over 23,000 metabolites were detected in untargeted metabolomic analysis and 61 metabolites were significantly different between the two groups. HCA revealed 8 metabolite clusters containing metabolites largely upregulated in patients with TB disease, including anti-TB drugs, glutamate, choline derivatives, *Mycobacterium tuberculosis*-derived cell wall glycolipids (trehalose-6-mycolate and phosphatidylinositol) and pro-resolving lipid mediators of inflammation, known to stimulate resolution, efferocytosis and microbial killing. The resolvins were confirmed to be RvD1, aspirin-triggered RvD1, and RvD2. This study shows that high-resolution metabolomic analysis can differentiate patients with active TB disease from their asymptomatic household contacts. Specific metabolites upregulated in the plasma of patients with active TB disease, including *Mtb*-derived glycolipids and resolvins, have potential as biomarkers and may reveal pathways involved in TB disease pathogenesis and resolution.

## Introduction

The global burden of tuberculosis (TB) is vast, with an estimated 8.6 million new TB cases and 1.3 million deaths due to the disease in 2012 [1], [2]. Challenges in global TB control include human immunodeficiency virus (HIV)/TB co-infection and lack of effective vaccines and point of care diagnostics [3]. Also, there are no well-validated or specific biomarkers that can predict transition from latent TB to active TB disease or are useful to monitor efficacy of anti-TB drugs [4], [5]. Additionally, the rising global presence of multidrug resistant TB (MDR-TB) strains of *Mycobacterium tuberculosis (Mtb)* constitutes a public health crisis [1]–[3].

Metabolomics analysis incorporates nuclear magnetic resonance (NMR) spectroscopy-based or mass spectrometry (MS)-based technologies to identify hundreds to thousands of small-molecule metabolites in biofluids or tissues, coupled with biostatistics and bioinformatics to identify potential regulated biomarkers and metabolic pathways associated with disease [6]–[8]. Targeted or untargeted NMR- or MS-based metabolomics methods have recently been shown to distinguish the presence of specific infectious diseases, to predict therapeutic responses to anti-microbial agents, and to explore host-pathogen metabolic interactions, including in malaria [9], [10], chronic *Pseudomonas aeruginosa* pulmonary infection [11], HIV [12], and sepsis [13].

Targeted metabolomics methods have been used to characterize specific metabolites and regulated metabolic pathways endogenous to *Mtb* itself in cell culture studies [14]–[17] and *Mtb* metabolites possibly involved in drug resistance [18]. Metabolomics approaches have identified metabolic profiles associated with *Mtb* disease in animal models using NMR [19], [20] and in human latent TB infection or active TB disease using NMR-based analysis of serum [21] or MS-based analysis of sputum [22] or serum [23], [24].

We designed the current proof-of-principle study to determine the potential utility of high-resolution metabolomics to distinguish adults with newly diagnosed pulmonary TB compared to their matched household contacts and to determine potential metabolic features that may reflect host-*Mtb* metabolic interactions.

## Materials and Methods

### Ethics Statement

This study was approved by the Institutional Review Board of Emory University (Atlanta, GA, USA) and the Georgian National Center for Tuberculosis and Lung Disease (NCTBLD) Ethics Committee (Tbilisi, Georgia). All subjects provided written informed consent for participation in the study.

### Study Participants

Study participants for this ancillary metabolomics study were selected from a double blind, randomized, controlled, prospective trial of high-dose cholecalciferol treatment of patients with pulmonary TB disease (clinicaltrials.gov identifier NCT00918086) [25]. Inclusion criteria for patients were age ≥18 years, newly diagnosed TB as determined by a positive acid-fast bacilli (AFB) sputum smear, and later confirmed by a positive sputum culture for *Mtb* (performed at the Georgian National TB Reference Laboratory [NRL]) [26], ≤7 days of treatment with anti-TB drug therapy. Exclusion criteria included >7 days of anti-TB therapy (life time), hypercalcemia, nephrolithiasis, hyperparathyroidism, sarcoidosis, history of organ transplant, liver cirrhosis, requirement of hemodialysis, cancer in past 5 years, seizures, current pregnancy or lactation, serum creatinine >250 mmol/L, corticosteroid use in the past 30 days, current use of cytotoxic or immunosuppressive drugs, and current incarceration. Inclusion criteria for household contacts (controls) included lack of symptoms suggestive of TB disease or any other acute illness and documented negative sputum smear and culture. We chose a convenience sample of 17 TB subjects that had data available for their matched household contact.

### Sputum Culture, Drug Susceptibility Testing and First-Line Anti-TB Drug Therapy

Two sputum specimens were obtained from each patient subsequently confirmed to have active pulmonary TB disease. Direct sputum smears with Ziehl-Neelsen staining were examined by light microscopy. All sputum samples were sent to the NRL for culture on Löwenstein-Jensen (LJ)-based solid media, using standard methodologies as previously described [26], [27]. Positive cultures were confirmed to be *Mtb* complex using phenotypic tests and the MTBDR*plus* assay, as outlined elsewhere [26]. First-line anti-TB drugs (isoniazid, rifampicin, pyrazinamide and ethambutol) were given to all TB disease subjects ≤ one week prior to the plasma sampling. Drug susceptibility testing (DST) was done using absolute concentration method and for second-line drugs with proportion method on solid media with standard methodology, as previously described [27].

To compare individuals with TB disease to those without evident TB disease, we also obtained blood from 17 asymptomatic household contacts of all studied TB disease subjects who accompanied the index TB patient to clinic on the baseline visit (typically a close relative) and were documented to be sputum *Mtb* culture negative. None of these individuals received anti-TB drugs.

### Plasma Sample Collection

Peripheral blood samples were obtained by venipuncture from all 17 subjects with TB disease and well as an asymptomatic household contact without TB disease. Blood was collected in ethylenediaminetetraacetic acid (EDTA)-containing tubes, centrifuged and isolated plasma immediately stored frozen at −80°C. Samples were subsequently shipped on dry ice from Tbilisi to Emory University, Atlanta, GA, USA. Samples were never previously thawed, remained frozen during transit, and were frozen at −80°C in Atlanta prior to metabolomics analysis.

### Macronutrient Intake and Body Mass Index Assessment

Mean daily dietary intake of macronutrients (total calories, protein, fat and carbohydrate) was estimated in the three days prior to the blood collection using a validated culture-specific nutrient intake assessment instrument using the Nutrition Data System for Research software, version 2011, as previously described [25], [28]. Body mass index [BMI; body weight (kg)/height (m^2^)] was calculated at entry in all subjects using data obtained from a calibrated research stadiometer and digital body weight scale system (Tanita Inc; Arlington Heights, Illinois, USA).

### Metabolomics Analysis

#### High-Resolution Metabolomics

Thawed plasma (65 µL) was treated with 130 µl acetonitrile (2∶1, v/v) containing an internal isotopic standard mixture (3.5 µL/sample), as previously described [29]. Briefly, the internal standard mix for quality control consisted of 14 stable isotopic chemicals covering a broad range of chemical properties represented in small molecules [29]. Samples were mixed and placed in ice for 30 min prior to centrifugation for 10 min (16,100× g at 4°C) to remove protein. The supernatants (10 µL), for each high-resolution LC-MS analysis were then loaded onto an autosampler maintained at 4°C and analyzed in triplicate using a LTQ-Velos Orbitrap mass spectrometer (Thermo Scientific, San Jose, CA, USA) and C18 chromatography (Higgins Analytical, Targa, Mountain View, CA, USA, 2.1×10 cm). Elution was obtained with a formic acid/acetonitrile gradient [29] at a flow rate of 0.35 ml/min for the initial 6 min and 0.5 ml/min for the remaining 4 min. The first 2-min period consisted of 5% solution A [2% (v/v) formic acid in water], 60% water, 35% acetonitrile, followed by a 4-min linear gradient to 5% solution A, 0% water, 95% acetonitrile. The final 4-min period was maintained at 5% solution A, 95% acetonitrile. The mass spectrometer was set to collect data from mass/charge ratio (m/z) 85 to 2000 daltons over the 10-minute chromatography period. Electrospray ionization was used in positive ion mode for detection, as outlined [29], [30].

#### Tandem Mass Spectrometry

Glutamate (m/z 148.0594) and Mtb-derived trehalose-6-mycolate (m/z 801.5767) were verified by tandem MS/MS and fragmentation of co-eluting authentic reference standards added to plasma and prepared for analysis as described previously [29], [30]. Trehalose-6-mycolate for verification studies was purified from a specific Mtb strain (Colorado State University Mycobacteria Research Laboratory, Fort Collins, CO). Identity of specific D-series resolvins [resolvin D1 (RvD1), 7S, 8R,17S-trihydroxy-4Z, 9E, 11E, 13Z, 15E, 19Z-docosahexaenoic acid), resolvin D2 (RvD2), 7S, 16R, 17S-trihydroxy-4Z, 8E, 10Z, 12E, 14E,19Z- docosahexaenoic acid) and the aspirin-triggered RvD1 (AT-RvD1), 7S, 8R,17R-trihydroxy-4Z, 9E, 11E, 13Z, 15E, 19Z-docosahexaenoic acid] was confirmed using lipid mediator metabololipidomics analytical methods, as described by Serhan and colleagues [31], [32]. Briefly, five deuterium-labeled internal standards (0.5 ng) were added to plasma aliquots [d_5_-RvD2, d_8_-5-hydroxyeicosatetraenoic acid (d_8_-5-HETE), d_4_-leukotriene B_4_ (d_4_-LTB_4_), d_5_-lipoxin A_4_ (d_5_-LXA_4_) and d_4_-prostaglandin E_2_ (d_4_-PGE_2_) to facilitate quantification of mediator recovery. Samples were extracted using SPE columns, eluted with methyl formate, and organic solvent evaporated using a nitrogen stream. Samples were suspended in methanol for analysis by liquid chromatography coupled with tandem mass spectroscopy (LC-MS/MS), using QTrap ABI 5500 (ABSciex, Framingham, MA) [31], [33]. To monitor and quantify levels of the SPMs derived from arachidonic acid, docosahexaenoic acid (DHA) and eicosapentaenoic acid (EPA) in plasma samples [34], [35], multiple reaction monitoring (MRM) for signature ion fragments was performed with identification accomplished using LC retention time (RT) and ≥6 diagnostic ions of MS/MS spectrum. Quantification was determined based on peak MRM transition area and linear calibration curves [35].

#### Data Collection and Processing for High-Resolution Metabolomics

LTQ-Velos Orbitrap MS data were continuously collected over the 10-min chromatographic separation period and stored as.Raw files. The.Raw files were converted to.cdf format using Xcalibur file converter software (Thermo Fisher, San Diego, CA) and used for data extraction. Peak extraction and integration were performed using apLCMS with xMSanalyzer 1.0 [8], [36]. apLCMS (http://www.sph.emory.edu/apLCMS) is an adaptive processing software package designed for high resolution LC-MS data that performs data filtering, peak detection, and alignment and generates a feature table, where a feature is defined as the measured m/z, RT, and integrated ion intensity. We define a metabolite as any chemical in a biological system, where some metabolites may be gene-directed or environmental chemicals [6]. xMSanalyzer enhances the feature detection process by performing systematic data re-extraction and combining results from different parameter settings ([36]; http://sourceforge.net/projects/xmsanalyzer/).

### Statistical Analysis

Descriptive statistics for demographic and clinical data were performed. Two-tailed t-tests and two-tailed Fisher exact tests were used to compare metabolomics results from patients with TB disease and their household contacts using SAS version 9.3 (Cary, NC, USA) for continuous and categorical data, respectively. LIMMA, a package within the R framework for differential expression analysis, was used to identify differentially expressed metabolites between TB cases and household contacts [37]. To account for multiple comparisons, *P* values were adjusted using the Hochberg and Benjamini false discovery rate (FDR) at q = 0.05 to distinguish statistically significant metabolites that differed between the two groups [38]. HCA was performed using the differentially expressed metabolites to visualize patterns and detect clusters of co-regulated metabolites by disease state [6], [39]. An untargeted metabolome-wide association study (MWAS) based on the Pearson correlation analysis of the differentially expressed metabolites with all detected metabolites in plasma of subjects with TB disease and HCA was performed to understand the global association pattern of the discriminatory metabolites [6]. The statistical significance of correlations was determined using the Student's t-test method and visualized using a Manhattan plot where the x-axis corresponds to the metabolites (85–2000 *m/z*) and the y-axis corresponds to the negative log_10_ of the p-value [40], [41]. Targeted MWAS were also performed with *m/z* matching to the anti-TB drugs rifampin and ethambutol differentially expressed in TB disease versus HC subjects. FDR, HCA and Pearson correlations analyses were performed using R [8], [36]. Principal component analysis was performed using the pcaMethods R package (http://www.bioconductor.org/packages/release/bioc/html/pcaMethods.html). Support vector machine analysis was performed using the e1071 R package at default settings.

### Metabolite Annotation and Pathway Analysis

Putative metabolite identification of the discriminatory ions between TB and household contact subjects were determined using the open-access Metlin metabolite database (http://metlin.scripps.edu/) [42]. An in-house R package (manuscript in preparation) incorporating a suite of major small molecule databases [KEGG [43] http://www.genome.jp/kegg/), Human Metabolome Database (HMDB) [44], MetaCyc (http://www.metacyc.org), and ChemSpider (http://www.chemspider.com/)] was used to enhance reliability of ion annotation matching high-resolution *m/z* to metabolites in Metlin [45]. Pathway analysis was performed using KEGG [43].

## Results

### Subject Characteristics

The mean age (35±12 years) and sex of the 17 TB disease subjects were similar to the 17 household contacts studied (**Table 1**). All subjects were Caucasian; there were no differences between the two groups in terms of annual income and maximum educational level attained (not shown). The individuals with TB disease consumed significantly greater total daily calories and similar daily protein and fat intake per kg of body weight daily compared to household contacts. Despite higher daily caloric intake, the average body mass index (BMI) in the TB disease cohort was significantly lower than in matched housemates, reflecting the catabolic nature of TB disease [3], [28].

**Table 1:** Demographic characteristics of individuals with TB disease and their asymptomatic household contacts.

Characteristic^a^	TB Disease (n = 17)	Household Contacts (n = 17)	P-value
Age [years; mean (SD)]	35 (12)	42 (11)	0.09
Male sex, n (%)	10 (59%)	6 (35%)	0.16
Current smoker, n (%)	13 (76%)	7 (41%)	0.036
Currently employed, n (%)	8 (47%)	11 (64%)	0.023
Total calorie intake [kcal/kg/day; mean (SD)]	56.6 (17.8)	42.1 (11.7)	0.009
Total protein intake [g/kg/day; mean (SD)]	1.7 (0.8)	1.2 (0.5)	0.058
Total fat intake [g/kg/day; mean (SD)]	2.0 (0.8)	1.5 (0.5)	0.07
Total carbohydrate intake [g/kg/day; mean (SD)]	8.3 (2.4)	5.5 (1.4)	<0.001
BMI [kg/m^2^; mean (SD)]	20.7 (2.0)	25.6 (4.0)	<0.001

a Annual income and educational level were similar between the two groups (data not shown).

SD = standard deviation; BMI = body mass index; kcal/kg/day = kilocalories/kilogram/day; g/kg/day = gram/kilogram/day.

### High-Resolution Metabolomics Data

**Figure 1:**
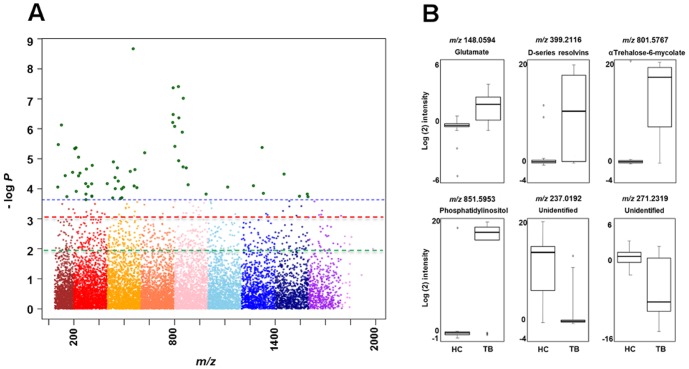
Plasma metabolome-wide association study (MWAS) of pulmonary tuberculosis (TB) disease in adults. (A) The Manhattan plot depicts the -log *P* analysis of 23,241 metabolites comparing 17 adults with newly diagnosed pulmonary TB disease and 17 asymptomatic adult household contacts who were sputum smear and culture negative for *Mtb*. The x-axis represents the *m/z* of the metabolites, ordered in increasing value from left (85) to right (2000). A total of 61 metabolites significantly differed [false discovery rate (FDR) q = 0.05] between the two groups (metabolites depicted above the horizontal dashed blue line). Metabolites above the horizontal dashed red line (n = 122) distinguished the TB disease and household contact cohorts at FDR q = 0.10, while metabolites above the dashed green line (n = 711) distinguished the two groups at FDR q = 0.20. (B) Box-and-whisker plots of log_2_ intensities comparing individuals with TB disease and household contacts for selected metabolites, with *m/z* and putative metabolite identification from Metlin and KEGG (left to right upper panel: glutamate, D-series resolvin, and trehalose-6-mycolate, respectively; left to right lower panel: phosphatidylinositol, and two unidentified metabolites that did not match to known metabolites in the databases, respectively).

Extraction of mass spectral data derived from C18 chromatography yielded 23,241 metabolites. Statistical analysis of the 34 subjects (FDR q = 0.05) demonstrated 61 metabolites distinguishing TB disease subjects from the household contacts. **Figure 1A** depicts a Manhattan plot charting the –log *P* value for each differentiating *m/z*. **Table S1 in File S1** lists these significantly different metabolites with *m/z*, respective RT, relative log intensities, median coefficient of variation, and negative log *P* value.

**Figure 1B** shows box and whisker plots of log_2_ intensities for six selected significant metabolites comparing the median and interquartile range for metabolite intensities in the household contacts (HC; left boxplot within each individual metabolite) and TB disease subjects (right boxplot within each individual metabolite). The individual *m/z* and putative metabolite identifications from Metlin are shown. The upper panel shows selected metabolites that were increased in TB subjects: from left to right, the amino acid glutamate, a D-series resolvin [31], [33], [34], [46], and the *Mtb*-specific cell wall glycolipid trehalose-6-mycolate [47], [48], respectively. The lower panel shows intensities of the metabolite matching phosphatidylinositol (PI), a key phospholipid present the cell wall of mycobacteria [47], [48] that was increased in TB subjects. This *m/z* matched to six putative PI molecules of different carbon chain lengths. The lower panel also shows two unidentified metabolites that were decreased in TB subjects relative to HC.

### Hierarchical cluster analysis and principal component analysis of plasma metabolites differentiating individuals with pulmonary TB disease from household contacts

**Figure 2:**
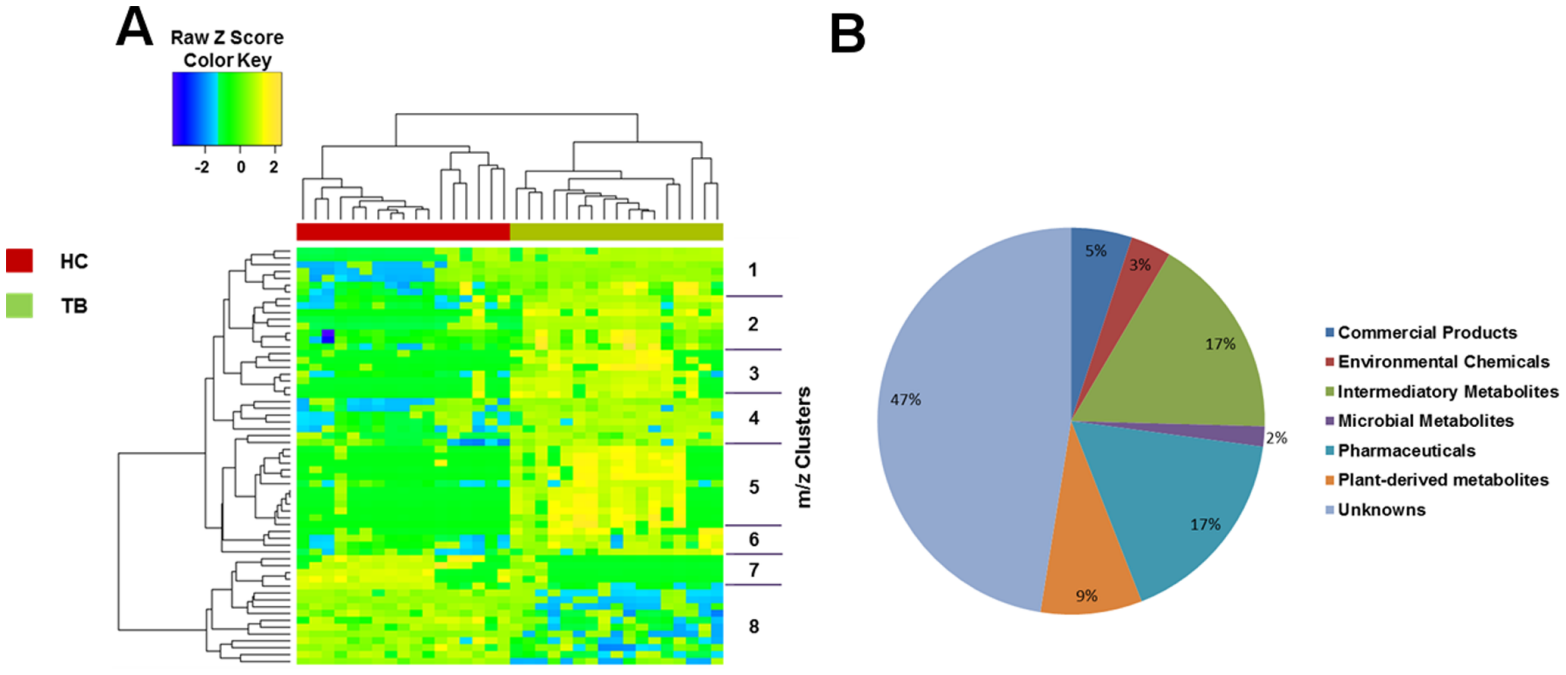
Significant metabolites that distinguish TB patients from household contacts. (A) Two-way hierarchical cluster analysis (HCA) using C18 chromatography shows 8 clusters of metabolites from human plasma and illustrates the patterns distinguishing those with active TB from household contacts without evidence of TB disease. The 17 subjects with TB disease (TB; shown in green) and the 17 household contacts (HC; shown in red) are shown along the x-axis. (B) Pie chart depicts chemical classes of the 61 significant metabolites from panel 2A according to high-resolution matches to metabolite databases [6].

Two-way hierarchical cluster analysis (HCA) of the 61 significant differentiating plasma metabolites is shown in **Figure 2A**. The pie chart (**Figure 2B**) depicts the distribution of the 61 differentiating metabolites within pan-metabolome categories [6]. Of interest, the largest category (47%) of this classification did not match to known metabolites in the Metlin metabolite database. As an alternative approach, we also performed principal component analysis (PCA; **Figure S1**) that showed a similar metabolite separation pattern between TB disease subjects and household contacts as seen with two-way hierarchical cluster analysis (HCA) (**Figure 1A**). The model was evaluated using the R2 and Q2 estimates (panel D). The model using the first two principal components suggests good predictive ability (PC1: R2 = 0.44, Q2 = 0.40; PC2: R2 = 0.7, Q2 = 0.64; PC3: R2 = 0.74, Q2 = 0.60) [49].

**Table S2 in File S1** lists the 59 metabolites with retention time (RT) >30 seconds grouped according to HCA cluster, and signifies cluster number, *m/z*, RT and putative metabolite match from Metlin [42]. As shown in the HCA heat map (**Figure 2A**), accurate mass metabolite matches within cluster 1 to 6 were all significantly increased in subjects with TB disease. In contrast, clusters 7 and 8 contained primarily unidentified (unmatched) metabolites, most of which were decreased in subjects with TB disease.

Metabolite cluster 5 contained *m/z* matches to the administered anti-TB drugs rifampin and ethambutol (both the Na^+^ and the H^+^ adducts of these agents were detected). The *m/z* for the administered anti-TB drugs isoniazid (*m/z* 120.0548) and pyrazinamide (*m/z* 124.0498) were detected at low intensity in some individuals with TB disease. Targeted MWAS to determine the association of *m/z* for rifampin and ethambutol with the full panel of detected metabolites demonstrated that these were highly correlated with the metabolites matching to isoniazid, pyrazinamide, and the isoniazid metabolites acetyl-hydrazine (*m/z* 97.0389) and isoniazid pyruvate (*m/z* 208.0682) [50], [51] (not shown). No anti-TB drug metabolite was detected in the household contacts.

### Verification studies for glutamate, D-series resolvins and trehalose-6-mycolate

**Figure 3:**
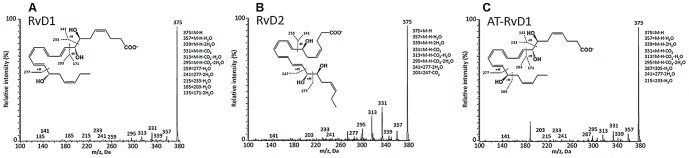
MS/MS fragmentation spectra show positive identification of resolvin D1 (RvD1), resolvin D2 (RvD2), and aspirin-triggered resolvin D1 (AT-RvD1) in plasma from subjects with TB disease. Metabololipidomics analytical methods that incorporated high-resolution liquid chromatography coupled with tandem mass spectroscopy (LC-MS/MS, ABI 5500, see methods) were used to verify these DHA-derived specialized pro-resolving lipid mediators [31], [33].

Tandem MS/MS and fragmentation of co-eluting authentic reference standards added to plasma was used to verify glutamate, D-series resolvins and trehalose 6-mycolate. One of the 61 significant plasma metabolites (*m/z* 399.2116) matched to several possible D-series resolvins using the Metlin database. We refined the annotation by using an in-house R package (manuscript in preparation), which matched the resolvin *m/z* to two specific resolvins, RvD1 and RvD2. As shown in **Figure 3**, verification studies confirmed the positive identification of RvD1, RvD2, and aspirin-triggered resolvin D1 (AT-RvD1) in the plasma from subjects with TB disease [31], [32]. **Figure S2** shows the presence of fragments 184.0736 and 86.0970 in both the reference standard and plasma sample at the same retention time and indicates the match to the [M-H_2_O+H] adduct for trehalose 6-mycolate is correct.

### Concomitant detection of putative Mtb cell wall-derived metabolites and the D-series resolvin metabolite in plasma of subjects with drug-susceptible or drug-resistant TB disease

Co-detection of the *m/z* matched to trehalose-6-mycolate, PI, and the D-series resolvins within study subject plasma (TB disease subjects with drug-susceptible TB, MDR-TB, and household contacts) is shown in **Table 2**. Notably, in all 13 patients with drug-susceptible TB with the detectable trehalose-6-mycolate metabolite in plasma, the PI metabolite was also concomitantly detected. Also, in all 12 patients with drug-susceptible TB with detectable plasma D-series resolvin(s) metabolite, both the trehalose-6-mycolate and the PI metabolites were detected. One of three patients with MDR-TB had concomitantly detectable D-series resolvin(s) and the trehalose-6-mycolate and PI metabolites. In another MDR-TB patient, the D-series resolvin(s) were demonstrated, but neither of the two *Mtb* cell wall-related metabolites was detected. The D-series resolvin(s), resolvin D1 and resolvin D2 were identified in the plasma of two household contacts, but was not linked to detection of either of the putative *Mtb* cell wall-related metabolites. One of the 17 household contacts demonstrated the trehalose-6-mycolate metabolite and PI concomitantly (**Table 2**).

**Table 2:** Linkage of trehalose-6-mycolate, phosphatidylinositol and the D-series resolvin metabolite(s) in plasma of patients without or with MDR-TB.

Metabolite feature	Non-MDR-TB patients^a^ ^b^	MDR-TB patients^c^	Household contacts^d^
Trehalose-6-mycolate	13/14	1/3	1/17
Phosphatidylinositol (PI)	14/14	1/3	1/17
D-series resolvin	12/14	2/3	2/17

a In all 13 non-MDR-TB patients with detectable trehalose-6-mycolate metabolite in plasma, the phosphatidylinositol metabolite was also detected.

b In all 12 non-MDR-TB patients with the D-series resolvin(s) metabolite detected in plasma, the trehalose-6-mycolate and PI metabolites were concomitantly detected.

c One of 3 patients with MDR-TB demonstrated the D-series resolvin(s), trehalose-6-mycolate and PI metabolites in plasma.

d One of the 17 smear-negative household contacts demonstrated both trehalose-6-mycolate and the PI metabolites in plasma, but these metabolites were undetectable the other household contacts.

The D-series resolvin metabolite(s) was detected in plasma of 2 other household contacts.

## Discussion

Our study demonstrates that discovery-based metabolomics profiling method can differentiate adults with pulmonary TB disease from their asymptomatic household contacts. The distinguishing metabolite profile includes specific resolvins, glutamate, and trehalose-6-mycolate, as well as other putative *Mtb* cell wall metabolites. A unique feature of our study compared to previous metabolomics studies in human TB is our use of liquid chromatography high-resolution mass spectrometry (LC-MS) metabolomics to profile >23,000 metabolites in plasma from humans with TB disease and concomitantly, their asymptomatic household contacts. Patients with active TB disease likely had similar exposures (e.g. specific dietary foods, environmental chemicals, household microbes, etc.) as the individual control subjects living in the same dwelling. Thus, specific metabolite differences between the two groups of subjects we report here were not likely to be due to other differences in the “exposome” of the respective groups [6] but rather were likely due to active *Mtb* disease and drug treatment.

Among the differentiating metabolic features were two of the four administered anti-TB drugs (rifampin and ethambutol). The *m/z* for isoniazid and pyrazinamide were detected at low intensity in some, but not all, individuals with TB disease. Metabolism and/or excretion of these agents and/or the sensitivity of our LC-MS method may explain why these latter drugs did not appear in the distinguishing metabolite panel. However, targeted MWAS showed the rifampin and ethambutol *m/z* were each highly correlated with metabolites matching to isoniazid, pyrazinamide, and the isoniazid metabolites acetyl-hydrazine and isoniazid pyruvate.

We also identified several metabolites not previously shown in plasma of patients with TB disease. Most notably, these included endogenously produced lipid mediators involved in the resolution of inflammation in a large number of conditions (specific D-series resolvins) [34], [35], and metabolites derived from the *Mtb* cell wall, including the mycobacterium specific cell wall glycolipid trehalose-6-mycolate and the non-specific cell wall phospholipid PI [47], [48], [52], [53]. In addition, many unidentified metabolites (unmatched in Metlin) were either increased or decreased in the plasma of the pulmonary TB disease subjects relative to controls, and may represent future targets for investigation [6]. This large proportion of unidentified metabolites is consistent with our previous studies using an earlier adaptation of this LC-MS method in several eukaryotic species, including man [7].

In our study, dietary intake does not seem to explain the overall metabolite profile that discriminated the two groups. It is possible, however, that the increased triglyceride metabolite (**Table S2 in File S1**) was related to higher recent fat or carbohydrate intake and the increased choline metabolite from increased intake of choline-rich foods such as eggs and meats (not shown). It is highly unlikely that modestly higher dietary protein intake in individuals with TB disease relative to the household contacts (**Table 1**) accounts for the single amino acid (glutamate) to be within the discriminating metabolite profile.

There is considerable promise for metabolomic analyses to advance understanding of TB and its pathogenesis. However, some of the limited previous metabolomic studies in human TB disease have lacked culture confirmation in all subjects, information on the course of disease after TB samples were collected, or dietary intake data [21]–[24]. Also, prior to the present study, the analytic platforms used for metabolomic analysis in individuals with TB disease measured a relatively small number of total metabolites (total metabolites = 30 to 498) [21]–[24]. Our similar results with both HCA and PCA confirm the accuracy of the significant metabolites to correctly classify the TB patients and household contact controls in this cohort.

Despite the limited coverage, considerable information has been gained from metabolomic analyses. For example, Weiner et al, using low-resolution MS and gas chromatography MS (GC-MS) methods, explored the metabolome of 428 distinct small molecules in a cross-sectional study in healthy controls, asymptomatic patients with latent TB, and patients with active TB disease [24]. A total of 20 metabolites differentiated subjects with TB disease (n = 44) compared to those with latent TB (n = 46) and healthy individuals (n = 46) combined. In subjects with TB disease, these included altered abundance of several amino acids, and increased N-acetylneuraminate, pyroglutamine (glutamate metabolism), inosine, and mannose [24].

Our data show for the first time *m/z* matches for two *Mtb* cell wall components in the plasma of patients with TB disease, the verified glycolipid trehalose-6-mycolate (a component of the mycolic acid layer of the cell wall) and PI. PI is a common component of cell wall membranes in both bacteria and humans and could be derived from various sources in these human subjects. However, these metabolites were detected concomitantly in 14 of 17 of the TB disease subjects at this similar early time point after clinical diagnosis of TB disease (**Table 2**). Mannose, which was upregulated in TB disease in the Weiner report [24], is a critical component of the *Mtb* cell wall glycan lipoarabinomannan (LAM) [47], [48], [54], [55] and is required for mycobacterial growth [56]. Relevant to our findings, in the *Mtb* cell wall LAM is anchored to both the mycolic acid layer and the cell membrane by PI [47], [48]. Therefore, it is possible that the increased mannose identified by Weiner et al. [24] was derived from cell walls of the infecting *Mtb*, consistent with our observations of the elevated metabolites matching to PI and trehalose-6-mycolate.

Previous metabolomic studies noted a variety of amino acid alterations that distinguished TB disease from controls [21], [23], [24]. We identified glutamate to be elevated in TB disease as the only discriminating amino acid (**Table S2 in File S1**), consistent with the upregulation of serum glutamate in TB disease noted by Zhou et al. [21] and, metabolically, with the decreased glutamine and elevated pyroglutamine noted by Weiner et al. [24]. Glutamate is a major amino acid utilized by *Mtb* during the growth phase [57] and this amino acid is a critical constituent for components of the *Mtb* cell wall [15], [58]. After granuloma formation in the host, *Mtb* shifts from a predominantly aerobic energy metabolism to anaerobic metabolism in the oxygen-limiting environment of the granuloma. Previous biochemical and targeted metabolomic studies have shown that varient tricarboxylic acid cycle pathways are employed by *Mtb* under hypoxic conditions, including a half-cycle to generate succinate from GABA that utilizes increased glutamate availability as a substrate [14], [17]. The C^5^ branched dibasic acid metabolic pathway is also utilized by *Mtb* to provide alternative carbon and nitrogen sources for energy, including glutamate [15], [59]. The upregulated glutamate in the differentiating plasma profile of this cohort of TB disease subjects may thus be a result of increased *Mtb* glutamate synthesis and represent a potential host-pathogen metabolic interaction.

This is the first study to identify an increase in specific resolvins in individuals with TB. Resolvins are lipid mediators derived from endogenous omega-3 fatty acids (DHA) from a genus of anti-inflammatory and specialized pro-resolving lipid mediators (SPM): resolvins, protectins, maresins, and lipoxins [32], [34], [35], [60]. Increased local production of D-series resolvins occurs during infection at sites of macrophage recruitment, particularly when macrophages are clearing apoptotic polymorphonuclear neutrophils (PMN) [31], [34], [61], [62]. D-series resolvins act as autocrine signals and facilitate phagocytosis and microbial killing by a variety of mechanisms. Apoptotic PMN themselves produce SPM and their uptake by macrophages (efferocytosis) during bacterial infection also stimulates macrophage synthesis of D-series resolvins, which, in turn, induce local anti-inflammatory effects and enhance microbial clearance [31], [34], [60], [61], [63]. RvD1 limits neutrophil-mediated tissue injury, acute inflammatory responses and trans-endothelial migration in inflammatory lung diseases in mice [63]. In a murine microbial sepsis model, RvD2 significantly dampened the local and systemic bacterial burden by preventing excessive leukocyte infiltration and enhancing clearance of microbes [62].

The upregulation of resolvins RvD1 and RvD2 in plasma of this small cohort of recently diagnosed TB disease patients likely reflects a host response to infection and tissue damage/inflammation (compared to the lack of this response in the apparently uninfected household contacts). To our knowledge, no publications have identified upregulation of any resolvin species in the blood of patients with non-TB lung diseases, although resolvin E1 was identified in the sputum of adult cystic fibrosis patients [64]. Our findings likely reflect a non-specific host homeostatic response, however, given that the circulating resolvins RvD1 and RvD2 are detectable in serum, plasma and lymphoid organs of healthy individuals and can be upregulated in blood, for example, by oral fish oil [32], [65]. Although the role of resolvins in *Mtb* infection is currently little understood, the results shown in **Table 2** suggest the possibility of a common pathophysiologic pathway in this cohort of TB disease subjects involving events that result in local generation of D-series resolvins in the lung granuloma that are detectable in plasma, concomitant with the presence of specific *Mtb* cell wall-related metabolites.

Limitations of this study include the cross-sectional nature, with single plasma samples obtained within the first week of drug therapy, the relatively small sample size and the few patients studied with MDR-TB. In addition, while the household contacts did not have active TB disease, no studies were carried out to assess whether they had latent TB infection. Further metabolomic studies are needed among patients with documented latent TB infection to detect potential biomarkers for transition to active disease. Another limitation of this pilot study is that we cannot explicitly determine relationships between identified metabolites and anti-TB therapy or TB progression. Also, our FDR controlling procedures exert less stringent control over false discovery compared to family wise error rate procedures (such as the Bonferroni correction); thus, we have consequently avoided over-interpretation of these data. As we had no *a priori* basis for a power calculation, this report should be viewed as hypothesis-generating and needs to be confirmed in a larger study. We did not obtain absolute identity of most of the discriminating metabolites by tandem MS/MS studies using authentic standards. Metabolites in plasma are an indirect measure of *Mtb* metabolism and local tissue response to infection; thus, metabolomics data from patient sputum or the *Mtb* organism itself would be of interest.

In summary, these pilot data show that adults with active TB disease can be differentiated from persons without active TB disease by high-resolution metabolic profiling of plasma. We were able to identify multiple metabolites relevant to *Mtb* disease (including *Mtb* cell wall components) and its unique metabolism, as well as D-series resolvins that may reflect a general response to TB disease in humans. Prospective and confirmatory studies are needed to gain pathophysiologic insight before and after initiation of conventional anti-TB drug therapy to better understand the significance of these novel findings. Of interest would be studies to determine whether plasma metabolic profiles can predict development of TB disease in individuals with latent TB, whether metabolite profiling can identify subjects with drug-susceptible versus MDR-TB, and the utility of these approaches and specific metabolomic signatures to predict responses to new anti-TB therapies including vaccines and newer drug combinations.

## Supporting Information

Figure S1 **Principal component analysis (PCA; panels A, B and C) showed a similar metabolite separation pattern between TB disease subjects and household contacts as seen with two-way hierarchical cluster analysis (HCA) (** **Figure 1A** **).** The model was evaluated using the R2 and Q2 estimates (panel D). The model using the first two principal components suggests good predictive ability (PC1: R2 = 0.44, Q2 = 0.40; PC2: R2 = 0.7, Q2 = 0.64; PC3: R2 = 0.74, Q2 = 0.60). Green squares = TB disease subjects (TB); Red squares = household contacts (HC).
(TIF)

Figure S2 **MS/MS fragmentation spectra show positive identification of trehalose 6-mycolate.** Authentic standard trehalose-6-mycolate was purified from *Mtb* at the Colorado State University Mycobacteria Research Laboratory, Fort Collins, CO. MS/MS spectra for *m/z* 801.5727 at collision energy of 35% for 1 uM trehalose 6-mycolate (A) and plasma (B). Presence of fragments 184.0736 and 86.0970 in both the reference standard and plasma sample at the same retention time indicate the match to the [M-H_2_O+H] adduct for trehalose 6-mycolate is correct.
(TIF)

Text S1 **This file contains all 61 significant** ***m/z*** **, retention times, minimum and maximum** ***m/z*** **, number of peaks, median coefficient of variation, and an internal scoring system (QRscore) with intensities for all samples included in this study.**
(TXT)

File S1 **Supporting Tables.**
(DOCX)
