# Supplementary figures and images for: Plasma Metabolomics in Human Pulmonary Tuberculosis Disease: A Pilot Study
Source: PLoS One. 2014 Oct 15;9(10):e108854. doi: 10.1371/journal.pone.0108854 (PMC4198093; doi:10.1371/journal.pone.0108854)

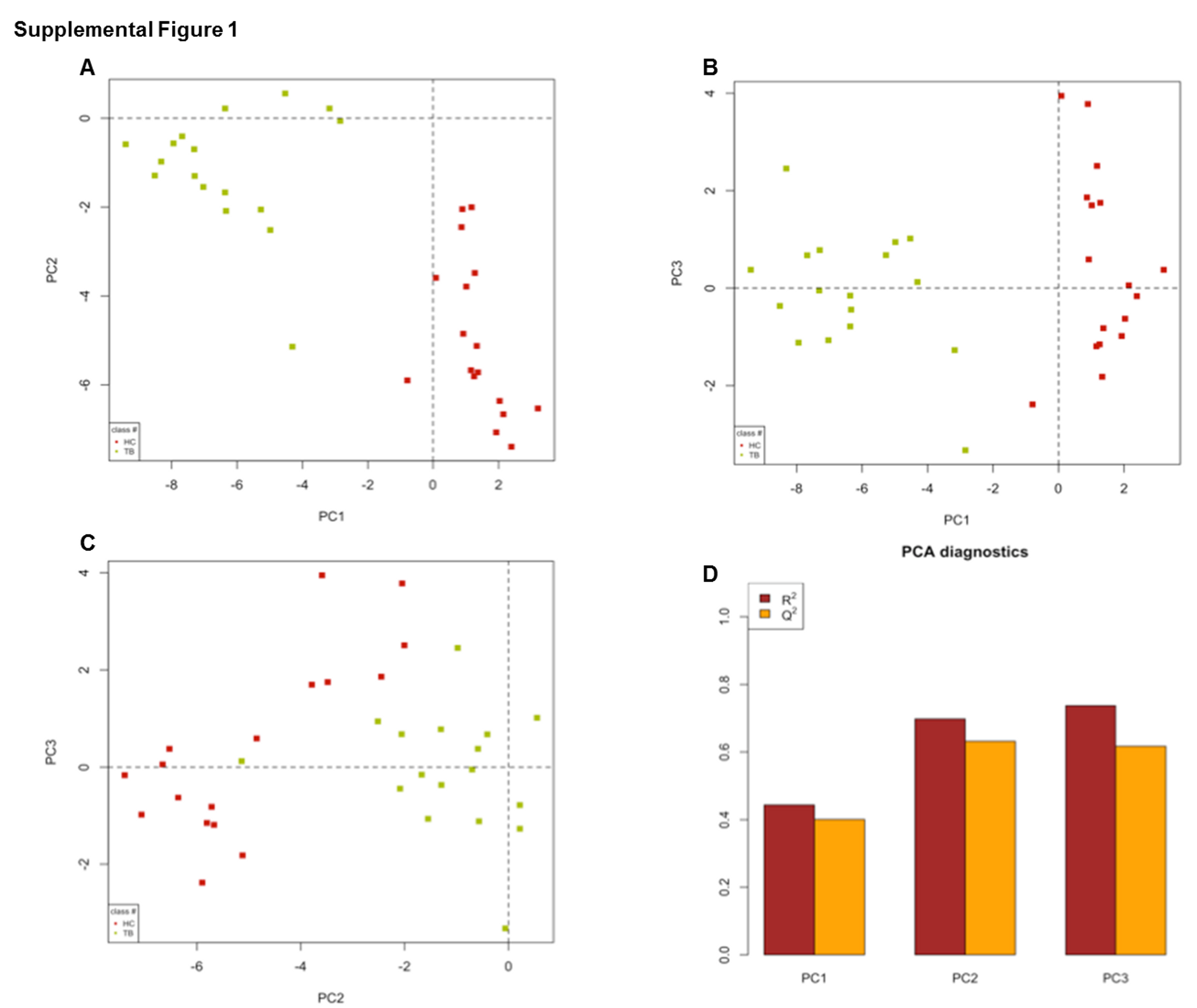

Supplement: Figure S1 — Principal component analysis (PCA; panels A, B and C) showed a similar metabolite separation pattern between TB disease subjects and household contacts as seen with two-way hierarchical cluster analysis (HCA) ( Figure 1A ). The model was evaluated using the R2 and Q2 estimates (panel D). The model using the first two principal components suggests good predictive ability (PC1: R2 = 0.44, Q2 = 0.40; PC2: R2 = 0.7, Q2 = 0.64; PC3: R2 = 0.74, Q2 = 0.60). Green squares = TB disease subjects (TB); Red squares = household contacts (HC). (TIF) [file pone.0108854.s001.tif]

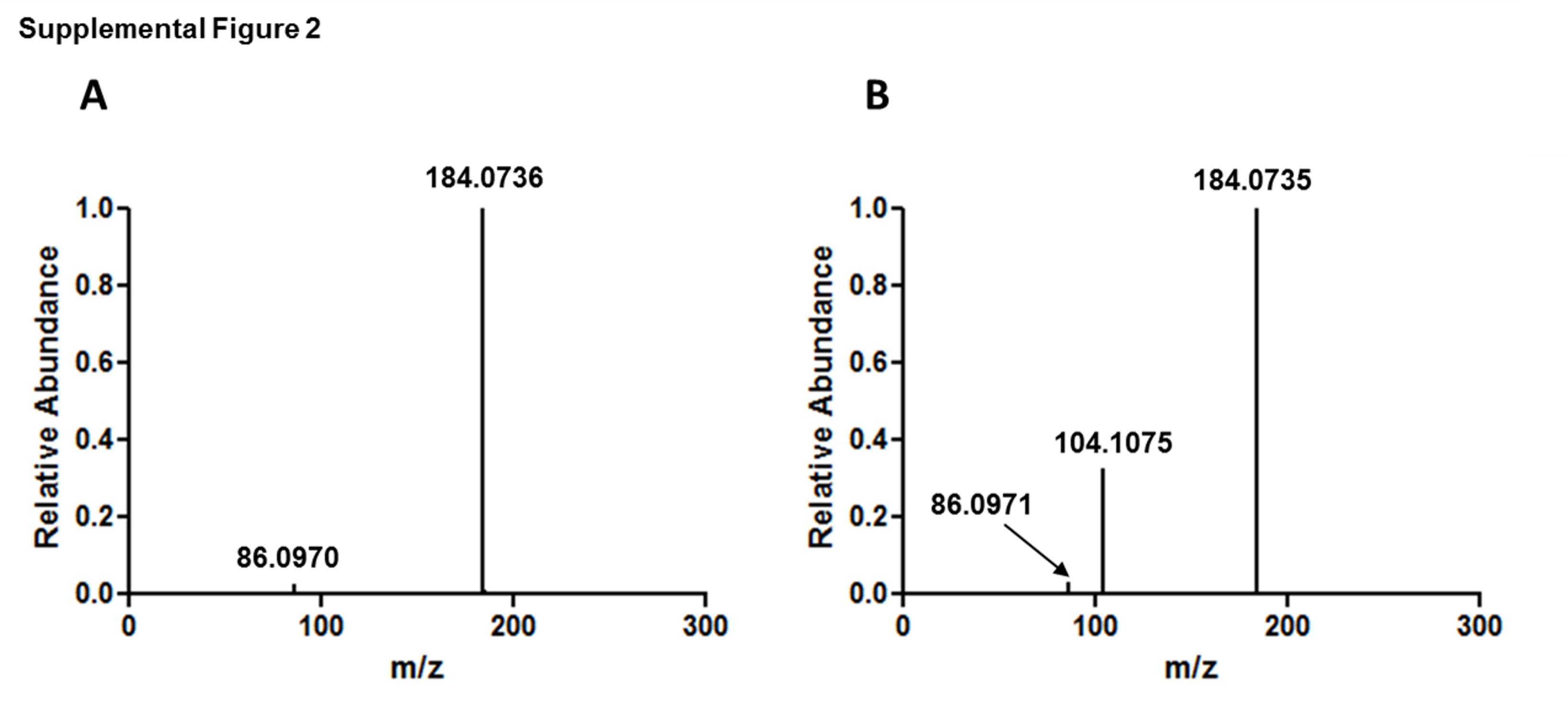

Supplement: Figure S2 — MS/MS fragmentation spectra show positive identification of trehalose 6-mycolate. Authentic standard trehalose-6-mycolate was purified from Mtb at the Colorado State University Mycobacteria Research Laboratory, Fort Collins, CO. MS/MS spectra for m/z 801.5727 at collision energy of 35% for 1 uM trehalose 6-mycolate (A) and plasma (B). Presence of fragments 184.0736 and 86.0970 in both the reference standard and plasma sample at the same retention time indicate the match to the [M-H2O+H] adduct for trehalose 6-mycolate is correct. (TIF) [file pone.0108854.s002.tif]
