# Supplementary material for: Plasma Metabolomics in Human Pulmonary Tuberculosis Disease: A Pilot Study
Source: PLoS One. 2014 Oct 15;9(10):e108854. doi: 10.1371/journal.pone.0108854 (PMC4198093; doi:10.1371/journal.pone.0108854)
Supplement: File S1 — Supporting Tables. (DOCX) [file pone.0108854.s004.docx]

**Online Supporting Material**

**Table S1. Metabolites Statistically Different Between TB Disease and HC Subjects^a^**

| ***m/z*** | **RT (sec)** | **Relative Log Intensities** | | **Median CV** | **-log *P*** |
| --- | --- | --- | --- | --- | --- |
|  |  | **HC** | **TB** |  |  |
| 104.1063 | 52 | -0.25 | 0.35 | 8.087 | 4.06 |
| 107.0232 | 64 | 0.55 | 10.85 | 23.15 | 5.47 |
| 125.0531 | 142 | 0.77 | 11.87 | 12.82 | 6.13 |
| 148.0594 | 71 | -0.76 | 1.52 | 114 | 4.44 |
| 158.8735 | 62 | 4.70 | -5.87 | 23.25 | 3.74 |
| 192.0231 | 50 | -8.61 | 0.51 | 19 | 4.55 |
| 205.1898 | 48 | -8.78 | 3.54 | 10.69 | 5.34 |
| 206.1934 | 47 | 0.71 | 11.71 | 7.518 | 5.34 |
| 212.9984 | 53 | -0.41 | 0.66 | 6.846 | 5.37 |
| 214.0050 | 48 | -0.89 | 1.36 | 10.94 | 3.92 |
| 227.1718 | 48 | 1.86 | 14.02 | 11.94 | 5.05 |
| 228.1751 | 47 | 1.04 | 10.72 | 16.46 | 4.43 |
| 237.0191 | 39 | 10.80 | 1.91 | 39.47 | 4.52 |
| 270.9566 | 56 | -0.59 | 0.45 | 6.427 | 4.17 |
| 271.2319 | 548 | 0.23 | -6.06 | 36.81 | 3.83 |
| 271.9629 | 50 | -7.41 | 0.92 | 22.02 | 3.64 |
| 273.9602 | 50 | 1.07 | 11.50 | 18.85 | 4.66 |
| 284.0310 | 79 | 10.46 | 1.55 | 17.88 | 4.08 |
| 305.1028 | 53 | -9.61 | -0.92 | 30.9 | 4.17 |
| 306.9330 | 53 | -7.94 | -0.41 | 42.47 | 3.75 |
| 307.2149 | 320 | 0.75 | -5.91 | 37.09 | 3.76 |
| 309.1807 | 132 | -5.45 | 3.72 | 18.23 | 4.78 |
| 399.2116 | 329 | 1.20 | 10.24 | 13.3 | 4.17 |
| 431.1446 | 84 | -3.79 | 5.99 | 8.974 | 3.70 |
| 435.2326 | 376 | 1.55 | -8.29 | 8.301 | 4.89 |
| 444.8320 | 52 | -7.79 | 0.36 | 26.85 | 4.37 |
| 446.8292 | 51 | -8.28 | 0.15 | 13.79 | 4.01 |
| 459.8740 | 51 | -8.32 | 0.37 | 42.63 | 4.70 |
| 464.1383 | 129 | 1.27 | 9.62 | 21.66 | 4.26 |
| 478.6161 | 57 | 12.19 | 2.41 | 11.62 | 3.68 |
| 484.9452 | 47 | -8.73 | 0.37 | 44.04 | 4.00 |
| 485.9535 | 51 | -11.08 | -1.46 | 19.36 | 3.70 |
| 495.2198 | 248 | -5.75 | 1.16 | 16.7 | 4.05 |
| 536.2780 | 378 | 0.38 | -9.01 | 5.93 | 4.58 |
| 553.9406 | 50 | -13.32 | 0.01 | 17.62 | 8.66 |
| 558.7576 | 53 | -0.52 | -9.86 | 47.99 | 4.10 |
| 564.4350 | 402 | -10.61 | 0.33 | 41.6 | 4.64 |
| 574.9516 | 51 | -9.44 | -0.63 | 35.12 | 4.03 |
| 621.9278 | 51 | -3.96 | 6.72 | 19.19 | 5.20 |
| 789.3655 | 272 | 0.47 | 11.67 | 9.716 | 6.21 |
| 791.3806 | 304 | 0.84 | 14.35 | 3.981 | 7.37 |
| 792.3868 | 304 | 0.67 | 12.84 | 7.124 | 6.47 |
| 800.5743 | 422 | 2.03 | 16.14 | 10.84 | 6.08 |
| 801.5767 | 446 | 1.20 | 13.30 | 39.19 | 5.42 |
| 823.4076 | 304 | 0.98 | 15.68 | 6.108 | 7.41 |
| 824.4129 | 303 | 1.04 | 13.06 | 4.121 | 4.94 |
| 825.5768 | 497 | 0.78 | 13.42 | 16.56 | 6.36 |
| 845.3897 | 304 | 0.02 | 10.37 | 5.492 | 5.89 |
| 850.5915 | 467 | -3.39 | 10.02 | 24.2 | 4.73 |
| 851.5953 | 471 | 1.13 | 15.14 | 10.86 | 7.02 |
| 869.8372 | 38 | -13.11 | -1.47 | 19.61 | 4.70 |
| 880.7371 | 45 | 11.63 | 1.64 | 21.14 | 4.13 |
| 988.8253 | 37 | -10.87 | -1.18 | 44 | 3.83 |
| 1117.1021 | 294 | 0.50 | -8.10 | 43.42 | 4.06 |
| 1321.9177 | 427 | 1.42 | 12.45 | 26.34 | 5.37 |
| 1329.4225 | 297 | 1.03 | -6.72 | 39.7 | 3.85 |
| 1453.0526 | 296 | 0.64 | -9.51 | 16.46 | 4.49 |
| 1544.5580 | 289 | 2.05 | -6.27 | 44.43 | 3.75 |
| 1591.6414 | 38 | -7.43 | 1.37 | 24.84 | 3.83 |

MDR-TB= multidrug resistant TB (resistance to both isoniazid and rifampin)

CV= coefficient of variation; HC= asymptomatic household contact of subject with TB disease;

*m/z*= mass/charge ratio; RT= retention time; sec=seconds, TB = tuberculosis

**^a^** False discovery rate (q=0.05)

**Table S2: Clusters and specific plasma metabolites that distinguish individuals with TB disease from household contacts**

| **Cluster** | ***m/z*** | **RT (sec)** | **Metlin Matches** |
| --- | --- | --- | --- |
| 1 | 988.8253 | 37 | Unidentified |
|  | 869.8372 | 38 | Triglyceride |
|  | 484.9452 | 47 | Quercetin |
|  | 446.8292 | 51 | Unidentified |
|  | 444.8320 | 52 | Unidentified (Cl^-^) |
|  | 270.9566 | 56 | Environmental exposure (cosmetic) (C12) |
|  | 212.9984 | 53 | Pesticide (Linuron) |
| 2 | 621.9278 | 51 | Unidentified |
|  | 192.0231 | 50 | Plant hormone |
|  | 273.9602 | 50 | Unidentified |
|  | 553.9406 | 50 | Unidentified |
|  | 485.9535 | 51 | Thyroid hormone mimetic |
|  | 214.0050 | 48 | Drug |
|  | 148.0594 | 71 | Glutamate |
|  | 104.1063 | 52 | Choline |
| 3 | 1321.9177 | 427 | Unidentified |
|  | 825.5768 | 497 | Unidentified |
|  | 399.2116 | 329 | D-series Resolvin |
|  | 850.5915 | 467 | Phosphatidylserine (C12) |
|  | 800.5743 | 422 | Phosphatidylserine (C12) |
|  | 851.5953 | 471 | Phosphatidylinositol (C13) |
|  | 801.5767 | 446 | *Mtb* cell wall compound (trehalose-6-mycolate) (C13) |
| 4 | 459.8740 | 51 | Herbicide (Bromofenoxim) |
|  | 271.9629 | 50 | Dichlorophenolindophenol (C13) |
|  | 574.9516 | 51 | Unidentified |
|  | 305.1028 | 53 | Chiral alcohol; acrylonitrile |
|  | 306.9330 | 53 | Unidentified |
|  | 564.4350 | 402 | Unidentified |
|  | 495.2198 | 248 | Unidentified Drug |
| 5 | 227.1718 | 48 | Ethambutol (Na^+^) |
|  | 206.1934 | 47 | Sphingosine |
|  | 228.1751 | 47 | Unidentified Drug |
|  | 125.0531 | 142 | Unidentified |
|  | 107.0232 | 64 | Unidentified |
|  | 205.1898 | 48 | Ethambutol (H^+^) |
|  | 823.4076 | 304 | Rifampin (H^+^) |
|  | 791.3806 | 304 | Unidentified (C12) |
|  | 792.3868 | 304 | Unidentified (C13) |
|  | 824.4129 | 303 | Unidentified |
|  | 789.3655 | 272 | Unidentified Drug |
|  | 845.3897 | 304 | Rifampin (Na^+^) |
| 6 | 464.1383 | 129 | Unidentified |
|  | 309.1807 | 132 | Androstenedione |
|  | 431.1446 | 84 | Unidentified Drug |
|  | 1591.6414 | 38 | Unidentified |
| 7 | 880.7371 | 45 | Unidentified |
|  | 237.0191 | 39 | Protein kinase inhibitor |
|  | 478.6161 | 57 | Unidentified |
|  | 284.0310 | 79 | Phosphotyrosine |
|  | 158.8735 | 62 | Unidentified |
|  | 558.7576 | 53 | Unidentified |
| 8 | 536.2780 | 378 | Vignatic acid A |
|  | 435.2326 | 376 | Plant toxin |
|  | 271.2319 | 548 | Unidentified |
|  | 1329.4225 | 297 | Unidentified |
|  | 1117.1021 | 294 | Unidentified |
|  | 1544.5580 | 289 | Unidentified |
|  | 307.2149 | 320 | Ruscopine |
|  | 1453.0526 | 296 | Unidentified |

Elements in parentheses represent different adducts of the same metabolite (e.g. rifampin).

*m/z* = mass/charge ratio; RT= retention time; sec=seconds.

Unidentified metabolites were those without a *m/z* match in Metlin.
